# Ochratoxin A and Citrinin Differentially Modulate Bovine Mammary Epithelial Cell Permeability and Innate Immune Function

**DOI:** 10.3390/toxins14090640

**Published:** 2022-09-16

**Authors:** Ran Xu, Umesh K. Shandilya, Alexandros Yiannikouris, Niel A. Karrow

**Affiliations:** 1 Department of Animal Biosciences, University of Guelph, Guelph, ON N1G 2W1, Canada; 2 Alltech Inc., Center for Animal Nutrigenomics and Applied Animal Nutrition, 3031 Catnip Hill Road, Nicholasville, KY 40356, USA

**Keywords:** bovine MAC-T cells, citrinin, epithelial paracellular permeability, in vitro cell culture, mycotoxins, ochratoxin A

## Abstract

Frequent detection of mycotoxins ochratoxin A (OTA) and citrinin (CIT) in ruminant feed and feedstuff can be a potential threat to feed safety, animal performance and health. Ineffective biodegradation of these mycotoxins by rumen microflora following ingestion of contaminated feeds can lead to their circulatory transport to tissues such as mammary gland as the result of their biodistribution throughout the body. The bovine mammary epithelium plays a pivotal role in maintaining milk yield and composition and contributes to innate immune defense of the udder. The present study is the first to investigate individual effects of OTA and CIT on barrier and innate immune functions of the bovine mammary epithelium using a bovine mammary epithelial cell line (MAC-T). Results indicated that OTA and CIT exposure for 48 h significantly decreased cell viability in a concentration-dependent manner (*p* < 0.05). A decrease in transepithelial electrical resistance and increase in paracellular flux of FITC-40 kDa dextran was significantly induced by OTA treatment (*p* < 0.05), but not by CIT after 48 h exposure. qPCR was performed for assessment of expression of tight-junction proteins, Toll-like receptor 4 (*TLR4*) and cytokines after 4, 24 and 48 h of exposure. Both OTA and CIT markedly downregulated expression of *claudin 3* and *occludin* (*p* < 0.05), whereas CIT did not affect *zonula occludens-1* expression. Expression of *TLR4* was significantly upregulated by OTA (*p* < 0.001) but downregulated by CIT (*p* < 0.05) at 48 h. Expression of *IL-6*, *TNF-a* and *TGF-β* was significantly upregulated by OTA (*p* < 0.05), whereas *IL-6* and *TGF-β* expression was downregulated by CIT (*p* < 0.01). These results suggest that OTA and CIT could potentially differentially modulate barrier and innate immune functions of mammary epithelium. The present study not only throws light on the individual toxicity of each mycotoxin on bovine mammary epithelium but also lays the foundation for future studies on the combined effects of the two mycotoxins.

## 1. Introduction

Mycotoxins are naturally produced as toxic secondary metabolites by various fungal species that grow on a variety of feed ingredients, therefore animal feeds under favorable environmental conditions worldwide. Co-occurrence of different mycotoxins commonly occurs in animal feed. Mycotoxin contamination challenges feed quality and safety, and has become one of the most significant hazards to the global feed supply chain [1]. The consumption of mycotoxin-contaminated feed by animals can lead to a variety of adverse effects on animal health and production, such as reduced productivity and fertility [2,3,4], and increased susceptibility to infectious diseases due to mycotoxin-induced compromised immune system [5,6]. Moreover, climate change has been predicted to increase the incidence and patterns of mycotoxin contamination [7,8,9], which could further accentuate their impact in agricultural production systems.

Ochratoxin A (OTA) is among the major mycotoxins that have aroused public concern due to its high toxicity and agri-economic significance [1,10]. OTA has been reportedly detected in cereal grains [11,12], silage [12,13], as well as livestock feeds for various species including dairy cattle [10,14,15,16] with the highest concentration of 305.6 μg/kg in dairy cattle feed in Turkey [15]. The mycotoxin citrinin (CIT) also deserves attention due to its known toxic effects in various mammalian species [17] and its frequent occurrence in agricultural commodities and animal feeds [17,18], commonly with OTA from the same fungal species (e.g. *Penicillium* and *Aspergillus*) [17,18,19]. Kelman et al. [20] reported maximum 81 μg/kg of CIT in the samples of Canadian forage for dairy cattle and goats. Data regarding the toxicity and the occurrence of CIT in food and feed however, are currently limited. The European Food Safety Authority [17] stated the need for more relevant CIT toxicity data to further refine risk assessment due to uncertainties in the current database. Neither of these two mycotoxins are currently regulated for ruminant feeds or feedstuff by Canadian Food Inspection Agency in Canada where this study was performed.

Ruminants are considered less sensitive to OTA than monogastric animals due to the microbial biotransformation of OTA in the rumen to less toxic ochratoxin α [19]. Similarly, CIT was also assumed to be highly degraded by rumen microflora, however, limited data are available regarding its overall toxic effects on ruminants [17]. Several factors can influence the rumen ecosystem such as rumen pH, rumen dysbiosis and redox potential [21,22], and these could compromise ruminal biodegradation of mycotoxins and thereby increasing their bioavailability to the systemic circulation [23].

Other factors may also affect sensitivity to mycotoxins. Some mycotoxins possess antimicrobial properties for example, which could affect microflora biotransformation processes [17,24]. Sensitivity is also affected by animal production stages [25], heath status [26,27,28] as well as their feed composition [29]. Mycotoxins that escape ruminal degradation could be distributed to different tissues via systemic circulation where they could possibly exert their toxic effects [27,30], and also partition into edible animal products [17,19,31,32]. The mammary gland for example, is a likely site of action for several mycotoxins and their metabolites as mycotoxin residues including OTA has been reportedly detected in ruminant milk [19,31,32].

Mammary epithelial cells (MECs) are one of the critical functional cellular components of the mammary gland. They maintain and facilitate blood-milk barrier (BMB) function and participate in innate immune defense against pathogen; as such, MECs function to maintain tissue homeostasis in addition to secreting milk protein. Integrity of the BMB formed by MECs is critical for sustaining optimal milk composition during lactation and in preventing uncontrolled exchange of components between blood and milk via paracellular transport [33,34]. Tight junction (TJ) proteins that connect adjacent MECs are determinants of paracellular transport [35]. Thus, disrupted expression of MEC TJ proteins by mycotoxins could lead to leakiness and changes in milk composition [34].

In addition to their role in BMB, MECs also play a critical role in the host innate immune response. In response to microbial invasion of the teat canal [36,37], MECs first recognize conserved pathogen-associated molecular patterns (PAMPs) via their pattern-recognition receptors (PRRs) such as the Toll-like receptors, and subsequently secrete cytokines and chemokines that initiate inflammation, an innate immune response that leads to the influx of professional immune cells to clear the invading pathogens [38,39,40,41].

Despite previous in vitro studies showing the toxic effects of OTA on paracellular permeability and immune function in various cell models [42,43,44,45], studies on the toxic effect of OTA and CIT on the mammary epithelium are limited. In this study, individual effects of OTA and CIT on above-mentioned two important functions of MECs were investigated. Cell viability was first assessed to determine the cytotoxic effects of OTA and CIT on MECs. Transepithelial electrical resistance (TEER) and paracellular tracer flux were then measured as indicators of altered paracellular permeability. Gene expression of various TJ proteins, cytokines, and toll-like receptor 4 (*TLR4)* was also evaluated to assess the immunomodulatory effects of these mycotoxins.

## 2. Results

### 2.1. Cytotoxic Effects of OTA and CIT

**Figure 1:**
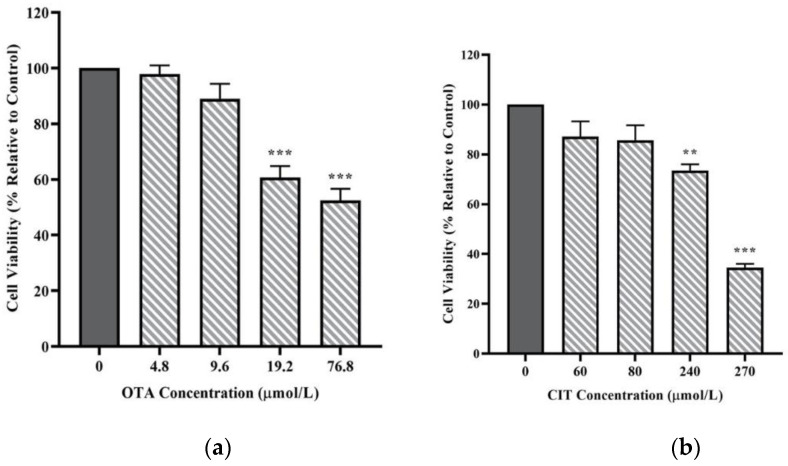
Effects of (**a**) Ochratoxin A (OTA) and (**b**) Citrinin (CIT) on MAC-T cell viability after 48 h exposure, respectively. Results are presented as the percentage of viable cells compared to the untreated control (0 μmol/L). Values are presented as the mean ± SEM of 3 independent experiments. Significant differences compared to control are indicated at *p* < 0.01 (**) and *p* < 0.001 (***).

Cell viability of MAC-T cells upon 48 h exposure to mycotoxin treatments was first measured to assess the cytotoxicity of mycotoxins. As shown in Figure 1, both OTA and CIT reduced viability of MAC-T cells in a concentration-dependent manner within the concentration range used in the present study. OTA and CIT significantly decreased cell viability at concentrations greater than 9.6 μmol/L (*p* < 0.001) and 80 μmol/L (*p* < 0.01), respectively. The calculated IC_50_ ± SE for OTA and CIT were 69.92 ± 18.35 μmol/L and 277.6 ± 44.56 μmol/L, respectively.

### 2.2. Effects of OTA and CIT on Paracellular Permeability

**Figure 2:**
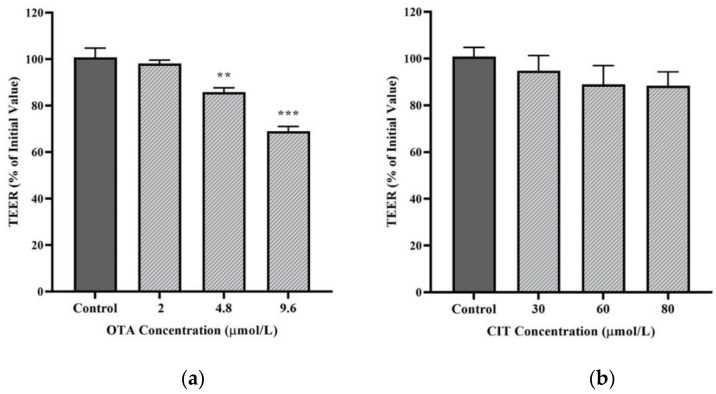
Effects of (**a**) Ochratoxin A (OTA) and (**b**) Citrinin (CIT) on transepithelial electrical resistance (TEER) of MAC-T cells after 48 h exposure, respectively. Results are presented as the percentage of TEER compared to the initial TEER values prior to mycotoxin treatment and values are presented as the mean ± SEM of 3 independent experiments. Significant differences compared to control are indicated at *p* < 0.01 (**) and *p* < 0.001 (***).

Transepithelial electrical resistance (TEER) and paracellular tracer flux were performed to assess the effects of OTA and CIT on paracellular permeability of MAC-T cell monolayer. As shown in Figure 2a, exposure to OTA for 48 h reduced TEER of MAC-T cell monolayer in a concentration-dependent manner. The TEER was not affected by 2 μmol/L OTA but was significantly reduced by OTA at higher concentrations of 4.8 μmol/L (*p* < 0.01) and 9.6 μmol/L (*p* < 0.001). In contrast, exposure to CIT for 48 h did not alter the TEER of MAC-T cell monolayer at any tested concentrations up to 80 μmol/L (*p >* 0.05) (Figure 2b). 

**Figure 3:**
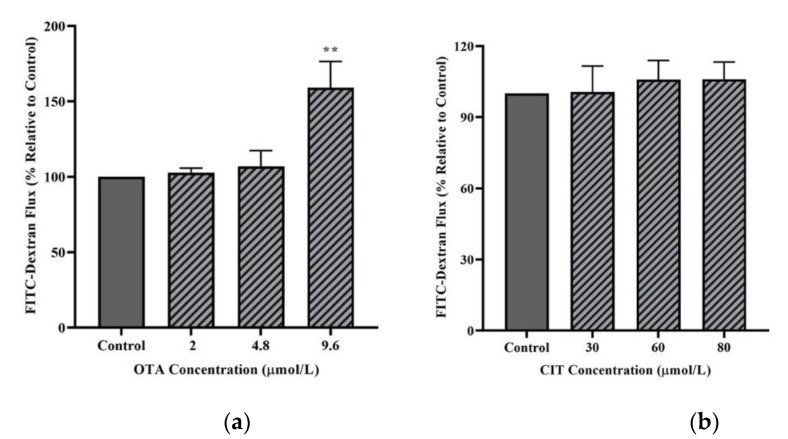
Effects of (**a**) Ochratoxin A (OTA) and (**b**) Citrinin (CIT) on paracellular flux of FITC-40 kDa dextran in MAC-T cells after 48 h exposure. Results are presented as the percentage of FITC-dextran flux compared to control groups (untreated) and values are the mean ± SEM of 3 independent. Significant differences compared to control (untreated) at are indicated at *p* < 0.01 (**).

With regards to paracellular flux (Figure 3), only the highest concentration of OTA (9.6 μmol/L) resulted in a significant increase in FITC-40 kDa dextran flux across MAC-T cell monolayer after 48 h exposure (*p* < 0.01), whereas 48 h exposure to CIT did not induce any significant changes in the permeability of MAC-T cells to dextran flux (*p >* 0.05). 

### 2.3. Gene Expression Analysis

**Figure 4:**
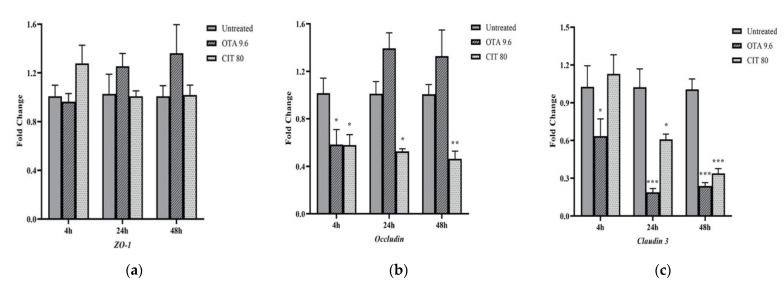
Effects of ochratoxin A at 9.6 μmol/L (OTA 9.6) and citrinin at 80 μmol/L (CIT 80) on mRNA expression of tight junction proteins (**a**) *Zonula Occludens-1* (ZO-1), (**b**) *Occludin* and (**c**) *Claudin 3* in MAC-T cells after 4, 24 and 48 h exposure, respectively. Results are presented as fold change and control groups (untreated) of each timepoint was used as calibrators; values are the mean ± SEM of 3 independent experiments. Significant differences compared to control (untreated) are indicated at *p* < 0.05 (*), *p* < 0.01 (**) and *p* < 0.001 (***), respectively.

The effects of mycotoxins on the relative mRNA expression of selected MAC-T cell TJ proteins *Zonula occludens-1 (ZO-1*), *claudin 3* and *occludin* were evaluated at three time points (4, 24 and 48 h) of mycotoxin exposure by qPCR. The highest concentration for each mycotoxin that did not exhibit cytotoxicity to MAC-T cells (9.6 μmol/L for OTA, 80 μmol/L for CIT) were used in this experiment. As shown in Figure 4, *ZO-1* was not significantly affected by either OTA or CIT at any timepoints (*p* > 0.05). In contrast, expression of *claudin 3* was significantly downregulated by OTA at all 3 timepoints (*p* < 0.05), and by CIT at 24 and 48 h (*p* < 0.05). The expression of *occludin* was also downregulated by OTA at 4 h (*p* < 0.05) and by CIT at all 3 timepoints (*p* < 0.05).

**Figure 5:**
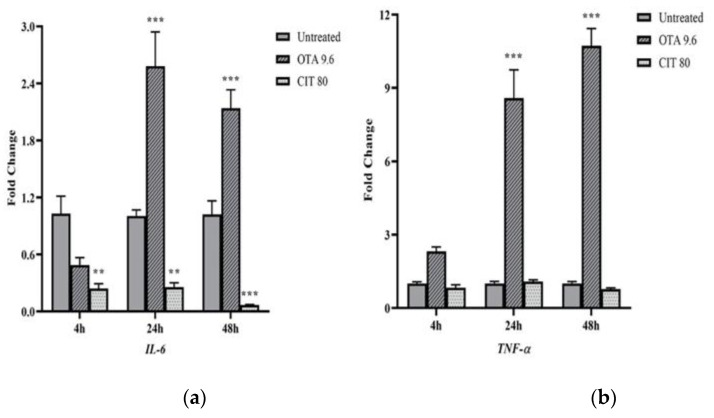

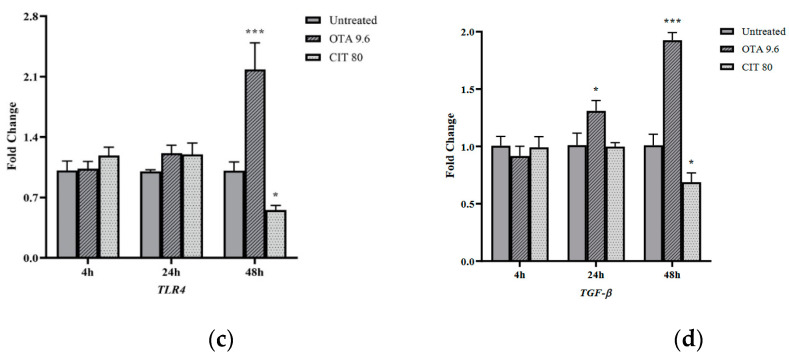
Effects of ochratoxin A at 9.6 μmol/L (OTA 9.6) and citrinin at 80 μmol/L (CIT 80) on mRNA expression of immune-related genes (**a**) Interleukin 6 (*IL-6)*, (**b**) Tumour necrosis factor alpha (*TNF-α),* (**c**) Toll-like receptor 4 (*TLR4)* and (**d**) Transforming growth factor beta (*TGF-β)* in MAC-T cells after 4, 24 and 48 h exposure, respectively. Results are presented as fold change and control groups (untreated) of each timepoint were used as calibrators; values are the mean ± SEM of 3 independent experiments. Significant differences compared to control (untreated) at *p* < 0.05 (*), *p* < 0.01 (**) and *p* < 0.001 (***), respectively.

Gene expression of immune-related genes was also quantified to assess the potential immunomodulatory effects of OTA and CIT. The results showed that OTA and CIT exhibited opposing immunomodulatory effects on MAC-T cells overall (Figure 5). Specifically, expression of *interleukin 6* (*IL-6*) (*p* < 0.001), *tumour necrosis factor alpha* (*TNF-α*) (*p* < 0.001) and *transforming growth factor beta* (*TGF-β*) (*p* < 0.05) were all upregulated by OTA at 24 and 48 h, but not affected at 4 h (*p >* 0.05). OTA also upregulated *TLR4* expression at 48 h (*p* < 0.001). In contrast, expression of *IL-6* was downregulated by CIT at all 3 timepoints (*p* < 0.01), but CIT did not significantly alter *TNF-α* expression at any timepoints (*p >* 0.05). Expression of *TGF-β* and *TLR4* was also downregulated by CIT at 48 h (*p* < 0.05).

## 3. Discussion

Udder health is of importance to the high-yield production of high-quality milk. As such, is required for profitability of dairy farmers, human food security and is a welfare issue. Sufficient high-quality milk and colostrum is also important for providing balanced nutrients and adequate passive immune protection to the newborn calves [46,47]. As a pressing issue to feed and livestock industry worldwide, the effects of mycotoxin contamination on bovine udder deserves more attention. Application of the MAC-T cell line has been well-documented in the literature as in vitro model to help investigate basic questions related to mammary gland physiology [48,49,50,51]. In vitro cell models have been widely accepted as effective approaches for mycotoxicity testing [52,53,54,55]. To our knowledge, the present study is the first parallel study to investigate individual toxicity of frequently detected mycotoxins OTA and CIT on paracellular permeability and innate immune functions of the bovine mammary epithelium.

Enhancing the production of high-quality milk is the ultimate goal of mammary gland in lactating dairy cow. Milk production is partly influenced by the number of MECs, the fundamental milk secretory units of mammary gland [56]. The integrity of BMB formed by MECs represents an important barrier between the interstitium and blood, and it is critical to milk production. Disruption of this barrier integrity has been associated with altered milk composition during lactation [34]. The mammary epithelium barrier also contributes to immunocompetence of the udder by protecting the mammary gland from microbial invasion and involving an innate immune response during intramammary infection. A compromised epithelium barrier has been associated with increased susceptibility of microbial translocation, reduced milk yield and quality [38,57,58,59,60].

In the current study, to assess the effects of OTA and CIT on cell viability, cell viability assay was performed using Calcein AM, a cell-permeant dye that has been frequently used to determine cell viability in eukaryotic cells [61,62,63]. We showed that OTA and CIT treatments significantly reduced viability of MAC-T cells after 48 h in a concentration-dependent manner. These findings are in line with previous in vitro studies reporting their cytotoxicity on various epithelial cells [43,64,65,66,67,68]. We also demonstrated that OTA was more toxic in reducing viability of MAC-T cells than CIT, as shown by the calculated IC_50_ of OTA (69.92 μmol/L) and CIT (277.6 μmol/L), which was consistent with previously reported toxicity differences between these two mycotoxins [65,69,70]. Collectively, our results suggested that exposure to OTA and CIT at certain concentrations could potentially lead to the loss of milk secreting cells in the udder during lactation and resulting impairment of BMBs by cell loss.

Blood-milk barrier function of MECs is critical to maternal lactogenesis, subsequent galactopoiesis and milk secretion [35,71]. The integrity of this barrier is primarily determined by TJ. Tight junction becomes highly impermeable to prevent transport of ion and small molecules via paracellular route during lactation in healthy cows [35,72], and opening of TJ during lactation lead to permeable BMB and changes in milk composition [34]. We first assessed transepithelial electrical resistance and paracellular tracer flux, two parameters to indicate paracellular permeability and epithelial barrier integrity. Decreased TEER or increased tracer flux is indicative of increased epithelial paracellular permeability. In this study, non-toxic concentrations (9.6 μmol/L for OTA, 80 μmol/L for CIT) were selected for each mycotoxin based on cell viability results in an attempt to exclude any changes in paracellular permeability caused by the damage of cell monolayer. A significant decrease in TEER of MAC-T cell monolayer was observed after 48 h of OTA treatment. Our results were in agreement with previous studies where OTA-induced reduction in TEER in human (Caco-2) and porcine (IPEC-J2) intestinal epithelial cell models has been extensively reported [42,73,74,75,76,77,78,79]. In contrast, CIT treatment did not significantly alter TEER values of MAC-T cell monolayer in this study. Among few studies on CIT- associated effects of epithelial permeability, Nakayama et al. [45] reported an increase in TEER in CMT93-II, a mouse rectum cell line induced by CIT at 125 μmol/L after 48 h exposure, which was inconsistent with our results. The discrepancy could be explained by different cell types originated from different species associated with species- and organ-specific mechanisms, and mycotoxin concentrations used in experimental designs, which were the contributing factors to variation in mycotoxin-induced effects on TEER reported in the literature [42,76,77,80,81,82,83]. In correlation with the TEER results in the present study, CIT did not affect paracellular flux of FITC-40 kDa across the MAC-T monolayer, suggesting unaffected paracellular permeability, which could be attributed to the lower concentrations used in the present study, as it was previously demonstrated that mycotoxin-induced effects in paracellular flux of FITC-labeled dextran could be concentration-dependent [43,76,79,82]. Whereas, we observed an increase in paracellular flux of FITC-40 kDa dextran after 48 h OTA treatment, which was in line with previously reported OTA-induced increase in paracellular tracer flux [42,43,78,79]. Collectively, our study demonstrated that exposure to OTA could disrupt barrier function of MECs by increasing its paracellular permeability, and this could result in an increased amount of OTA in the milk potentially leading to food safety issues.

We next investigated the mRNA expression of the TJ proteins (*ZO-1*, *occludin* and *claudin 3)* to assess the effects of OTA and CIT on epithelial integrity at the transcriptional level by performing qPCR analysis. Our results demonstrated that mRNA expression of *ZO-1*, *occludin* and *claudin 3* was differentially modulated in response to OTA and CIT exposure, respectively; *Claudin 3* and *occludin* was downregulated by both mycotoxin treatments, whereas *ZO-1* being unaffected. Such differential toxic effects observed on TJ proteins have also been previously reported for OTA [42,78,84] and other mycotoxins [59,85]. Claudin 3 is one of the members of a large claudin protein family. Claudins along with occludin are the two major transmembrane proteins that play a role in maintaining the TJ barrier function, and they are linked to the actin cytoskeleton via scaffolding proteins, such as ZO-1 [72]. Claudins have been considered as the key determinant of paracellular characteristics. They form the backbone of TJ and contribute to the tightness of TJ by sealing the paracellular pathway [45]. It is known that different claudins selectively modulate paracellular pathway [86,87]. Wang et al. [82] for example, found that Claudin 4 was the core TJ protein involved to Caco-2 cell permeability upon DON exposure. In contrast, occludin contributes to TJ stabilization and optimal barrier function [88]. Our results suggested that decreased expression of *claudin 3* could be the potential primary contributor to OTA-induced increase in paracellular permeability of MAC-T cells, whereas other claudin proteins might play a predominate role in responding to CIT treatment in MAC-T cells, as indicated by the results from TEER and tracer flux.

A successful host immune response is generally the results of a dynamic balance between pro- and anti-inflammatory elements, with the ultimate goal of clearing the pathogen and limiting host damage [89]. qPCR analysis of *TLR4, IL-6, TNF-α* *and TGF-β* was performed to investigate the effects of OTA and CIT on the innate immune response of the mammary epithelium. In the present study, we observed potential opposing effects of OTA and CIT on innate immune function of MECs. Both effects suggested that OTA and CIT could lead to aberrant immunity in mammary epithelium. Toll-like receptor 4 is of importance to mammary gland defense in that it is capable of recognizing lipopolysaccharide, the PAMP derived from Gram-negative mastitis-causing pathogens [90]. The observed OTA-induced upregulation and CIT-induced downregulation of *TLR4* expression at 48 h suggested possible immunostimulatory effect of OTA whereas immunosuppressive property of CIT. Consistent with our results, upregulated *TLR4* expression were previously reported in different cell models [91,92] as well as in duck liver [93]. However, studies on immunomodulatory effects of CIT are limited. In an in vivo study, Islam et al. [94] reported a downregulation of *TLR3* induced by CIT in mice spleen, but no effect observed for *TLR4*, suggesting CIT exposure may selectively affect toll-like receptors. 

We next evaluated mRNA expression of cytokines given their important roles in inflammatory response. Likewise, we observed possible immunostimulatory effect of OTA and immunosuppressive property of CIT, as shown by OTA-induced upregulation of *IL-6*, *TNF-α* and CIT-induced downregulation of *IL-6*. Consistently, previous studies also reported OTA upregulated mRNA expression of *IL-6* and *TNF*-α in cell models [44,92,95,96]. IL-6 and TNF-α are two well-known pro-inflammatory cytokines that can locally and systemically initiate the immune response in the host [60,97]. Whereas TGF-β functions as an anti-inflammatory cytokine to dampen the inflammation as part of host homeostatic mechanisms [98,99,100], since an exaggerated or protracted dysfunctional mammary innate immune response could have deleterious effects resulting in uncontrolled acute or chronic mastitis [90]. Elevated levels of IL-6, TNF-α and TGF-β have been found to be associated with experimentally-induced inflammation in the bovine mammary gland [98,101,102,103]. Taken together, our results suggested that OTA could potentially induce over-activated innate immune system in mammary epithelium, which could lead to various negative outcomes to the host [104]; whereas CIT exposure could potentially lead to a noninfectious cause of immunosuppression in mammary gland, which could decrease resistance of mammary gland to infection [105]. Such contradictory properties observed in the present study could also suggest the potential antagonistic effects of combination of these two mycotoxins, which has been reported previously [6,69,70].

## 4. Conclusions

Ochratoxin A and CIT have been frequently detected in ruminant feedstuff and feeds. Inefficient degradation of mycotoxins resulting from disrupted rumen environments can lead to mycotoxin encounter with mammary gland, where subsequently their toxic effects may disrupt homeostasis of MECs. In the present study, OTA was found to increase paracellular permeability of MAC-T cell monolayer accompanied with decreased gene expression of certain TJ proteins at tested concentrations. Ochratoxin A could also potentially exert stimulatory effect on innate immune function of MAC-T by elevating gene expression of *TLR4* and pro-inflammatory cytokines. Conversely, CIT exposure could potentially induce immunosuppressive effects by suppressing expression of *TLR4* and pro-inflammatory cytokines but did not appear to affect paracellular permeability of MAC-T cells at tested concentrations in this study. The present study not only throws light on the individual toxicity of each mycotoxin on bovine mammary epithelium but also lays the foundation for future studies on the combined effects of the two mycotoxins.

## 5. Materials and Methods

### 5.1. Chemicals

Purified OTA and CIT (Sigma-Aldrich, St. Louis, MO, USA) were dissolved in dimethyl sulfoxide (DMSO) to final stock concentrations of 5.0 mg/mL and stored at −20 °C until further dilution in complete cell culture medium for subsequent experiments. The designated mycotoxin concentrations used for each subsequent experiment described below were prepared by serial dilutions of the stock concentrations in cell culture medium.

### 5.2. Cell Culture

The bovine mammary epithelial cell line (MAC-T) was maintained in the cell culture medium containing Dulbecco’s Modified Eagle Medium (DMEM) supplemented with 4.0 mmol/L L-glutamine, 10% heat inactivated fetal bovine serum, 2.5% HEPES buffer (Invitrogen), 1% Penicillin/Streptomycin (100 units/mL of Penicillin and 100 μg/mL Streptomycin; Invitrogen) and 1 mM Sodium Pyruvate (Invitrogen) as previously described with slight modifications [61]. Cells were maintained in T75 flasks in a humidified incubator at 37  °C with 5% CO_2_ and passaged by trypsinization using TrypLE™ (Gibco # 12605036) when reaching 80% confluency.

### 5.3. Cytotoxicity Assay

MAC-T cells were seeded at 2 × 10^4^ cells per well in 96-well microplates. After 24 h of cell culture, OTA (0, 4.8, 9.6, 19.2 and 76.8 μmol/L) and CIT (0, 60, 80, 240 and 270 μmol/L) were administered to the cells for 48 h [43,65,106,107,108]. The range of exposure concentrations were selected based on [42,63,64,109,110] and our preliminary studies (data not shown). At the end of mycotoxin exposure, 200 μL of Calcein AM (Invitrogen, CA, USA), a cell-permeant fluorescent dye was added to each well at a final concentration of 2 μmol/L, and the cells were incubated at room temperature for 45 min according to the references with slight modifications [61,63]. The fluorescence intensity (FI) was measured using a microplate reader (BioTek Instruments, VT, USA) at excitation 498/emission 528 nm. The percentage of viable cells was calculated using the following formula:

% Cell viability = Mean (FI_498 Treated cells_ − FI _Blank_)/Mean (FI_498 Untreated cell_ − FI _Blank_) × 100 (1)

where FI_498 Treated cells_ is the FI obtained from mycotoxin-treated groups; FI_498 Untreated cells_ is FI obtained from groups without any mycotoxin treatment; FI _Blank_ is the background signal resulting from Calcein AM treated wells without cells. Mean is the average FI of three replicates.

The IC_50_ (the 50% inhibitory concentration) of each mycotoxin was also calculated by fitting the data to non-linear Hill’s equation (Equation 1) using GraphPad Prism version 8.2.1 for Windows (GraphPad Software).

(2) $$Ŷ=a+\frac{b-a}{\begin{array}{c} \left[1+{\left(\begin{array}{c} \frac{c}{X} \end{array}\right)}^{d}\right] \end{array}}$$

where Ŷ is the expected response at dosage *X*, *a* is the minimum asymptote or the response when dosage = 0, *b* = the maximum asymptote or the stabilized response for an infinite dosage, *c* is the dosage at which 50% of the subjects are expected to show the desired response (that is, the response halfway between the minimum response asymptote a and the maximum response asymptote *b*), and *d* is the slope at the steepest part of the curve, also known as the Hill slope [111]. According to these cell viability data, non-cytotoxic concentrations were chosen for subsequent experiments in an attempt to exclude any changes in experiment endpoints caused by the damage of cell monolayer [43].

### 5.4. Transepithelial Electrical Resistance (TEER) Measurement

An insert-based MAC-T cell culture system was established as previously described with slight modifications [50] to assess the paracellular permeability of MAC-T cell monolayer. MAC-T cells were seeded at 2.5 × 10^4^ cells per Transwell® insert (Corning® Transwell® #3470, 6.5 mm, 0.4 µm pore size) pre-coated with Type I collagen at 10 μg/cm^2^ (C3867, Sigma-Aldrich) according to the manufacturer’s instruction and grown for 33 days in the same medium described above to ensure stable transepithelial electrical resistance (TEER) readings were yield according to our preliminary studies (data not shown). Medium was refreshed for both apical (AP) and basal compartments (BL) of the Transwell® inserts every other day [82]. On day 33, the cells were exposed to the increasing non-cytotoxic concentrations of either OTA (0, 2, 4.8 and 9.6 μmol/L), or CIT (0, 30, 60 and 80 μmol/L) in the AP compartment for 48 h. The TEER readings were measured before the addition of mycotoxins (TEER_0_) and after 48 h mycotoxin exposure (TEER_48_) using a Millicell ERS-2 Voltohmmeter (EMD Millipore Corporation) according to the manufacturer’s instruction. The change in TEER was expressed as TEER_48_ to TEER_0_ ratio, which was calculated according to the following formula [112]:

% TEER = TEER_48_ (Ω • cm^2^)/TEER_0_(Ω • cm^2^) × 100 (3)

### 5.5. Permeability Tracer Flux Assay

The paracellular tracer fluorescein isothiocyanate (FITC)-40 kDa dextran (FD-40, Sigma-Aldrich) was used in the tracer flux assay as another indicator of paracellular permeability of MAC-T cell monolayer. A tracer working solution of a final concentration of 1 mg/mL was prepared by dissolving FITC-40 kDa dextran powder in DMEM/Nutrient Ham’s Mixture F-12 (F12). The insert-based cell culture system was established as described above. On day 33, when stable TEER readings were achieved, the MAC-T cells were challenged with mycotoxin treatments as described above. After 48 h of mycotoxin exposure, the tracer working solution was added to the AP compartment of the culture system with DMEM/F12 added to the BL compartment. As previously described [43,113,114], after 2 h incubation, the fluorescence intensity of FITC-40 kDa in the BL compartment was measured by a microplate reader (Agilent Technologies formally BioTek Instruments, Winooski, VT, USA) at 498/528 nm.

### 5.6. Quantitative Real-Time PCR Analysis 

MAC-T cells were cultured in 24-well plates in triplicate at a density of 1 × 10^5^ cells per well. After 4 h, 24 h and 48 h exposure to OTA (0, 9.6 μmol/L) or CIT (0, 80 μmol/L) at the highest non-cytotoxic concentrations, cells were lysed with TRIzol Reagent (Invitrogen) according to the manufacturer’s instructions and stored at −80 °C until further RNA extraction. Total RNA was extracted and purified using RNeasy Mini Kit (Qiagen) according to the manufacturer’s instructions. RNA samples with a A260 nm/A280 nm ratio between 1.9 and 2.1 were reverse-transcribed into complementary DNA (cDNA) using High-Capacity cDNA Reverse Transcription Kit (Applied Biosystems) according to the manufacturer’s instructions. Quantitative real-time PCR (qPCR) was performed using SsoAdvanced Universal SYBR Green Supermix (Bio-Rad) in a total reaction volume of 10 μL with a StepOne Plus instrument (Applied Biosystems). The polymerase was activated at 95 °C for 10 min and the PCR was performed for 40 cycles (95 °C for 15 s and 60 °C for 1 min). The relative levels of gene expression were calculated using ΔΔCt method [115]. Glyceraldehyde-3-phosphate dehydrogenase (*GAPDH*) and ubiquitously expressed prefoldin like chaperone (*UXT*) were used as reference genes to normalize the expression of the target gene transcripts. The gene-specific primers are listed in Table 1.

### 5.7. Statistical Analysis

Statistical analysis was performed using GraphPad Prism version 8.2.1 for Windows (GraphPad Software). A one-way analysis of variance (ANOVA) was performed followed by Dunnett’s post-hoc test for multiple mean comparisons for cell viability, TEER and paracellular tracer flux. A two-way ANOVA followed by Dunnett’s post-hoc test for multiple mean comparisons was performed for the qPCR analysis. The data are presented as mean ± SEM of three independent experiments conducted in triplicate, and *p* < 0.05 was considered statistically significant.

## Figures and Tables

**Table 1:** Details of primer sequences, PCR efficiency, amplicon length, accession number of the target and reference genes.

Gene ^1^	Primer Sequence 5′-3′	PCR Efficiency (%)	Amplicon Length (bp) ^2^	Accession No.
*GAPDH: F*	GATGGTGAAGGTCGGAGTGAAC	101.711	100	NM_001034034.2
*GAPDH: R*	GTCATTGATGGCGACGATGT			
*UXT: F*	TTGACACAGTGGTCCCAGAC	96.603	143	NM_001037471.2
*UXT: R*	CTTGGTGAGGTTGTCGCTGA			
*ZO-1: F*	GCGAAATGAGAAACAAGCACC	97.78	121	XM_024982012.1
*ZO-1: R*	ATGAGTTGAGTTGGGCAGGAC			
*Claudin 3: F*	AGGGACTGTGGATGAACTGC	108.701	128	NM_205801.2
*Claudin 3: R*	CAGTAGGATGGCGATGACG			
*Occludin: F*	GCCAGCATATTCCTTCTACCC	103.487	139	NM_001082433.2
*Occludin: R*	AAGAGTGGAGGCAACACAGG			
*IL-6: F*	GGCTCCCATGATTGTGGTAGTT	105.691	523	NM_173923.2
*IL-6: R*	GCCCAGTGGACAGGTTTCTG			
*TNF-* *α* *: F*	CGGTGGTGGGACTCGTATG	103.751	352	NM_173923.2
*TNF-* *α* *: R*	CTGGTTGTCTTCCAGCTTCACA			
*TGF-* *β* *: F*	CCTGAGCCAGAGGCGGACTAC	99.181	130	NM_001166068.1
*TGF-* *β* *: R*	GCTCGGACGTGTTGAAGAAC			
*TLR4: F*	GAACAGGTAGCCCAGACAGC	99.35	151	NM_174198.6
*TLR4: R*	AGGCCATGATACGGTTGAAG			

^1^ F = forward; R = reverse; ^2^ bp = base pair.

## Data Availability

The datasets used and analyzed during the current study available from the corresponding author upon request.
